# Supplementary material for: Screening Effective Antifungal Substances from the Bark and Leaves of Zanthoxylum avicennae by the Bioactivity-Guided Isolation Method
Source: Molecules. 2019 Nov 20;24(23):4207. doi: 10.3390/molecules24234207 (PMC6930455; doi:10.3390/molecules24234207)

Supplementary Materials

Screening Effective Antifungal Substances from the Bark and Leaves of *Zanthoxylum avicennae* by the Bioactivity-Guided Isolation Method

Yongtong Xiong[1, 2†], Guan Huang[1, 2†], Zongli Yao[1, 2], China Zhao[1, 2], Xiang zhu[1, 2], Qinglai Wu[1, 2*], Xudong Zhou[3*] and Junkai Li[1, 2*]

[1] School of Agriculture, Yangtze University, Jingzhou, 434025, China;

[2] Institute of Pesticides, Yangtze University, Jingzhou, 434025, China;

[3] TCM and Ethnomedicine Innovation & Development Laboratory, School of Pharmacy, Hunan University of Chinese Medicine, Changsha, Hunan 410208, China.

[†] Yongtong Xiong and Guan Huang contributed equally to this study.

Spectra of compounds

1. Xanthyletin (compound 1)

^{1}H NMR (400 MHz, DMSO-*d*$_6$): *δ* 7.93 (d, J = 9.6 Hz, 1H), 7.41 (s, 1H), 6.77 (s, 1H), 6.49 (d, J = 10.0 Hz, 1H), 6.27 (d, J = 9.6 Hz, 1H), 5.85 (d, J = 10.0 Hz, 1H), 1.42 (s, 6H).

^{1}H NMR (400 MHz, $CDCl_3$): *δ* 7.59 (d, J = 9.6 Hz,1H), 7.27 (s, 1H), 6.72 (s, 1H), 6.34 (d, J = 10.0 Hz, 1H), 6.22 (d, J = 9.6, Hz, 1H), 5.70 (d, J = 10.0 Hz, 1H), 1.47 (s, 6H).

^{13}C NMR (101 MHz, DMSO-*d*$_6$): *δ* 160.58, 156.55, 155.31, 144.70, 131.80, 126.01, 120.92, 118.61, 113.12, 113.10, 103.83, 78.14, 28.40.

^{13}C NMR (101 MHz, $CDCl_3$): *δ* 161.29, 156.86, 155.43, 143.42, 131.25, 124.79, 120.78, 118.55, 112.99, 112.73, 104.40, 77.75, 28.35.

HRMS: calcd for $C_{14}H_{12}O_3$ $[M+H]^+$: 229.0859, found 229.0857.

^{1}H NMR spectrum of compound 1 (DMSO-*d*$_6$):

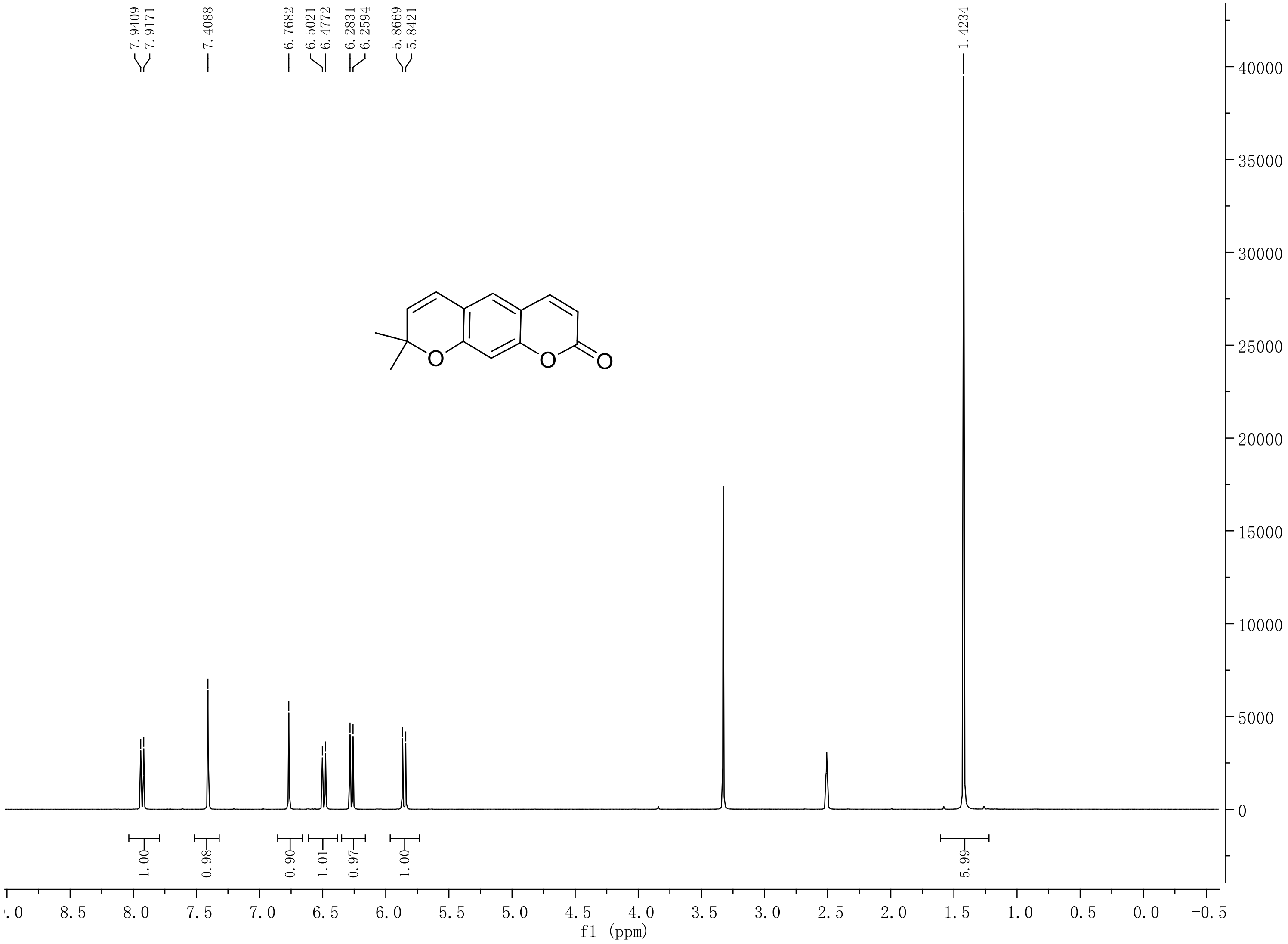
7.9409
7.9171
7.4088
6.7682
6.5021
6.4772
6.2831
6.2594
5.8669
5.8421
1.4234
40000
35000
30000
25000
20000
15000
10000
5000
0
O
O
O
1.00
0.98
0.90
1.01
0.97
1.00
5.99
8.5
8.0
7.5
7.0
6.5
6.0
5.5
5.0
4.5
4.0
3.5
3.0
2.5
2.0
1.5
1.0
0.5
0.0
-0.5
f1 (ppm)

^{13}C NMR spectrum of compound 1 (DMSO-d_6):

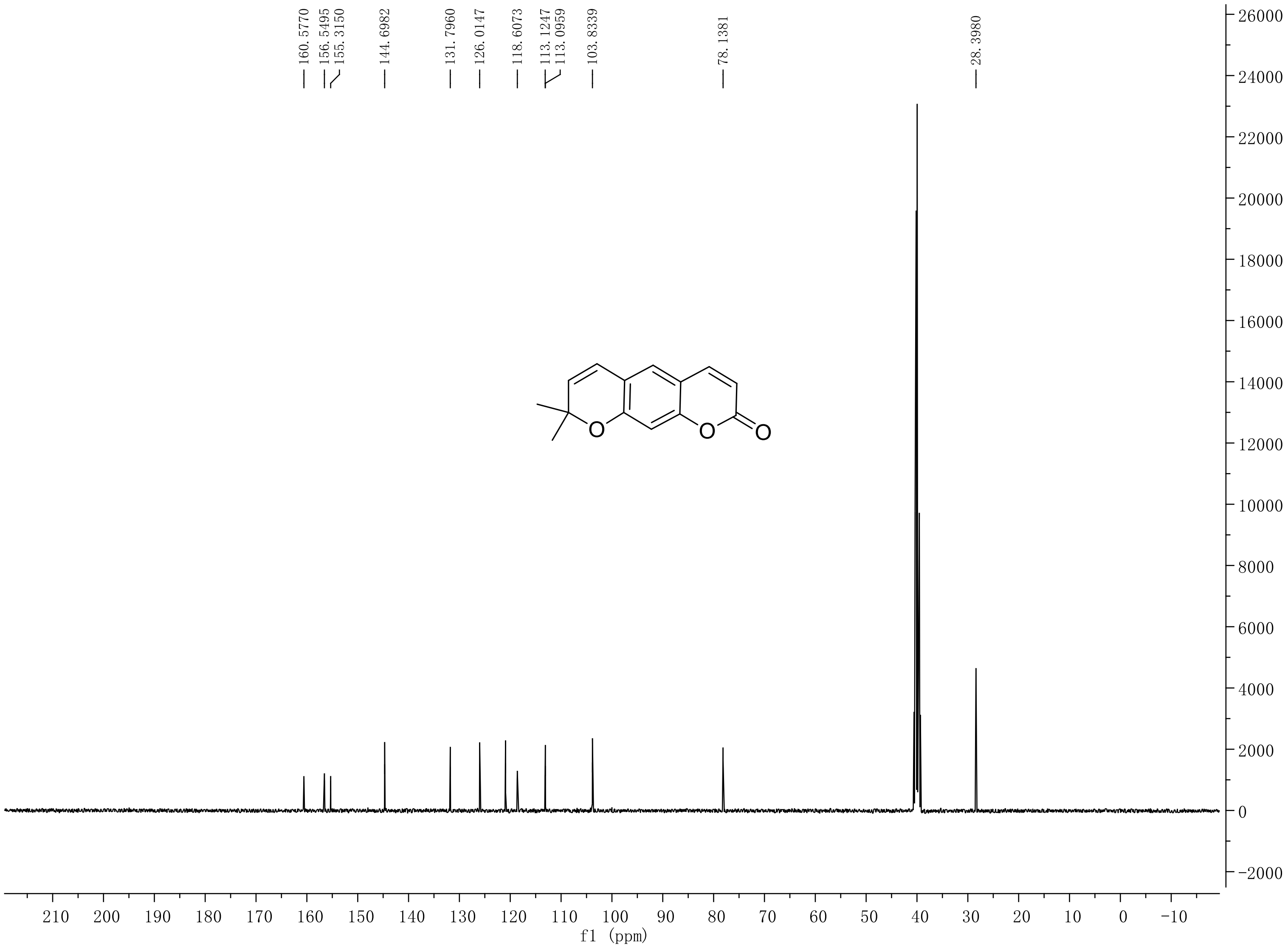

^{1}H NMR spectrum of compound 1 ($CDCl_3$):

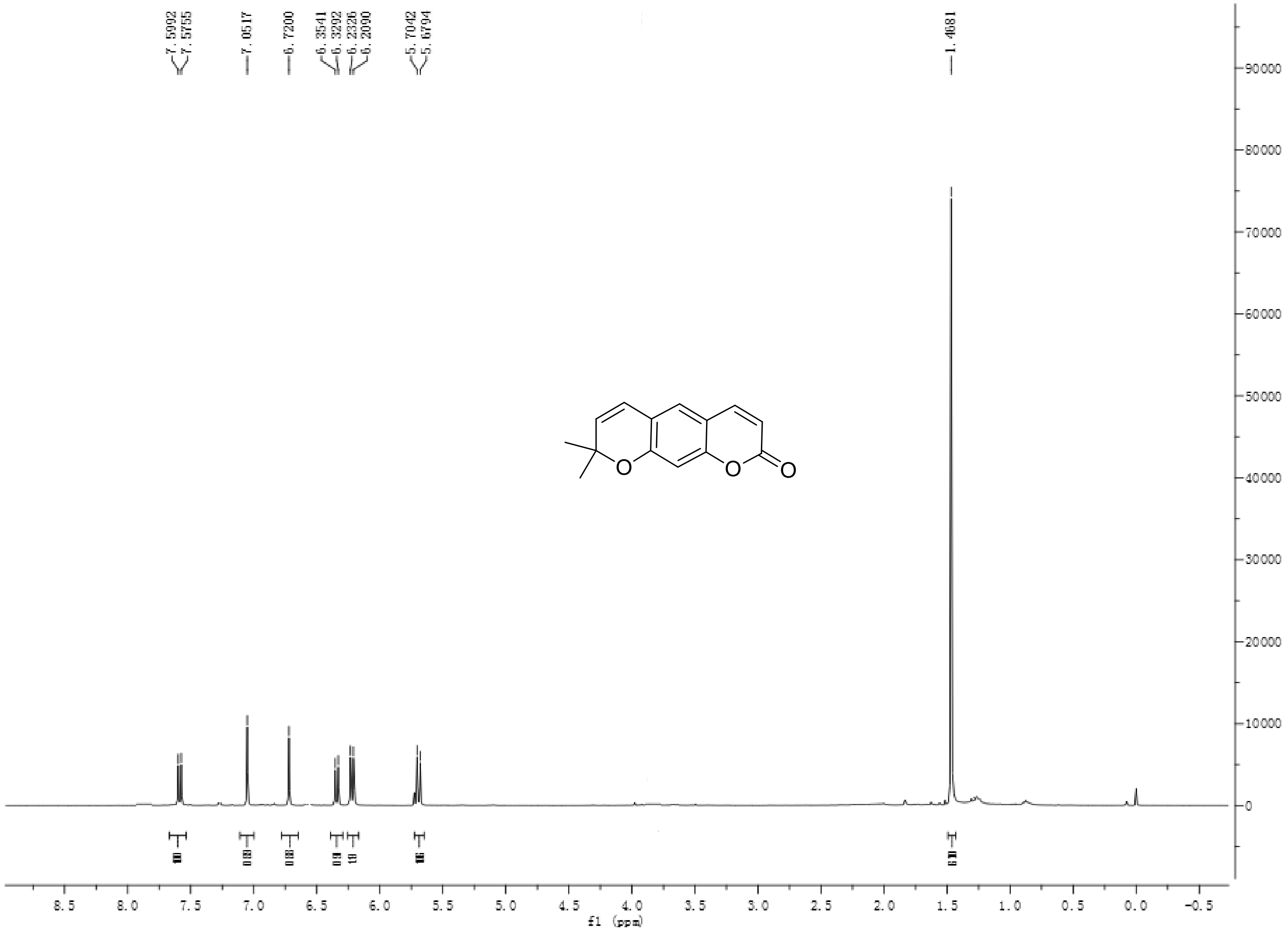

^{13}C NMR spectrum of compound 1 ($CDCl_3$):

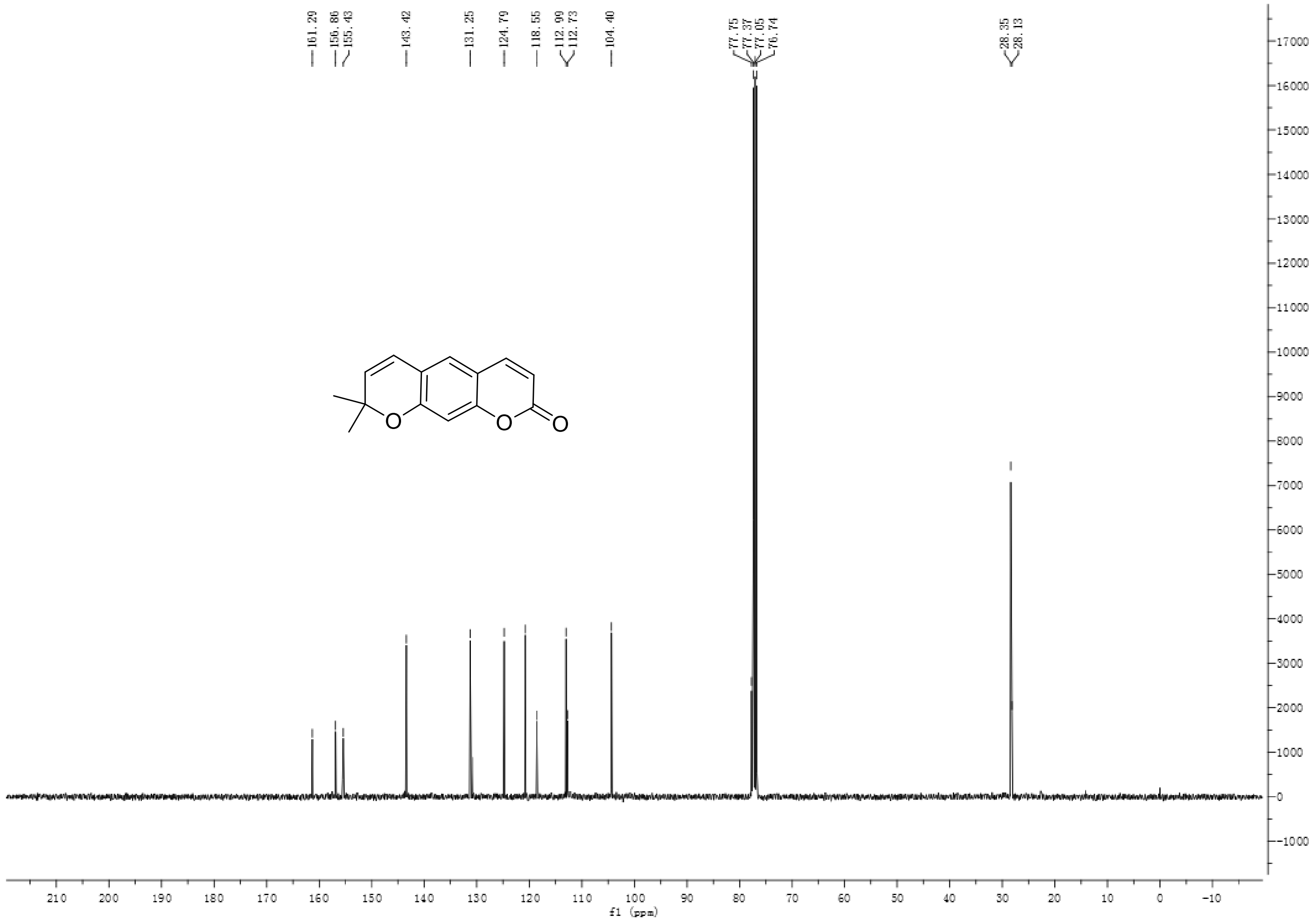

HRMS spectrum of compound 1:

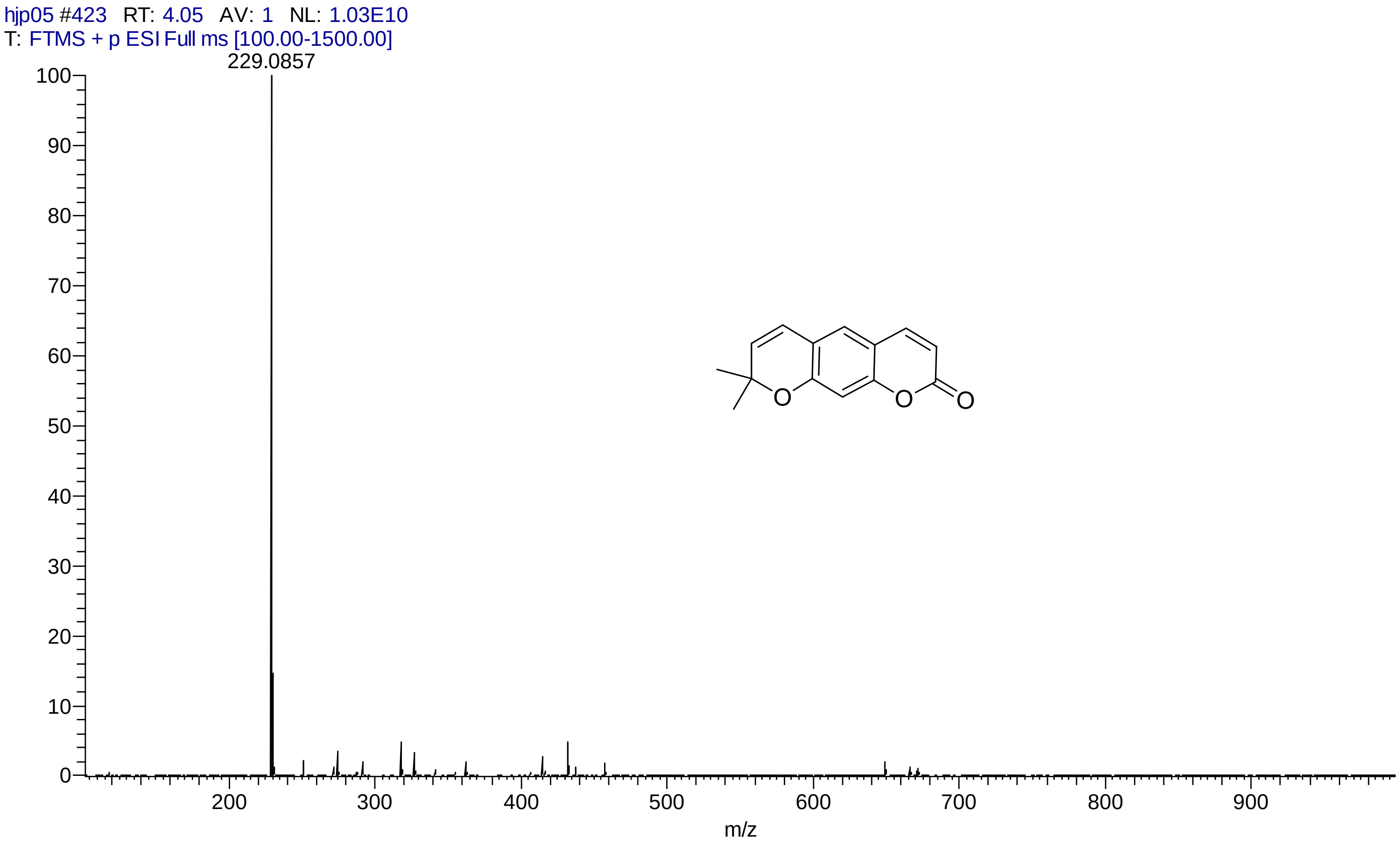

2. Luvangetin (compound 2)

^{1}H NMR (400 MHz, DMSO-d_6): δ 7.92 (d, J = 9.6 Hz, 1H), 7.18 (s, 1H), 6.48 (d, J = 10.0 Hz, 1H), 6.29 (d, J = 9.6 Hz, 1H), 5.88 (d, J = 10.0 Hz, 1H), 3.86 (s, 3H), 1.46 (s, 6H).

^{1}H NMR (400 MHz, $CDCl_3$): δ 7.59 (d, J = 9.6 Hz,1H), 7.27 (s, 1H), 6.72 (s, 1H), 6.34 (d, J = 10.0 Hz, 1H), 6.22 (d, J = 9.6, Hz, 1H), 5.70 (d, J = 10.0 Hz, 1H), 1.47 (s, 6H).

^{13}C NMR (101 MHz, DMSO-d_6): δ 160.22, 148.88, 148.01, 145.01, 135.21, 131.87, 121.23, 120.21, 119.25, 113.39, 113.26, 78.16, 77.35, 61.25, 28.18.

^{13}C NMR (101 MHz, $CDCl_3$): δ 160.64, 149.30, 148.29, 143.62, 135.64, 131.24, 121.09, 119.12, 119.10, 113.23, 112.99, 77.81, 77.25, 61.41, 28.21.

HRMS: calcd for $C_{15}H_{14}O_4$ $[M+H]^+$: 259.0965, found 259.0961.

^{1}H NMR spectrum of compound 2 (DMSO-d_6):

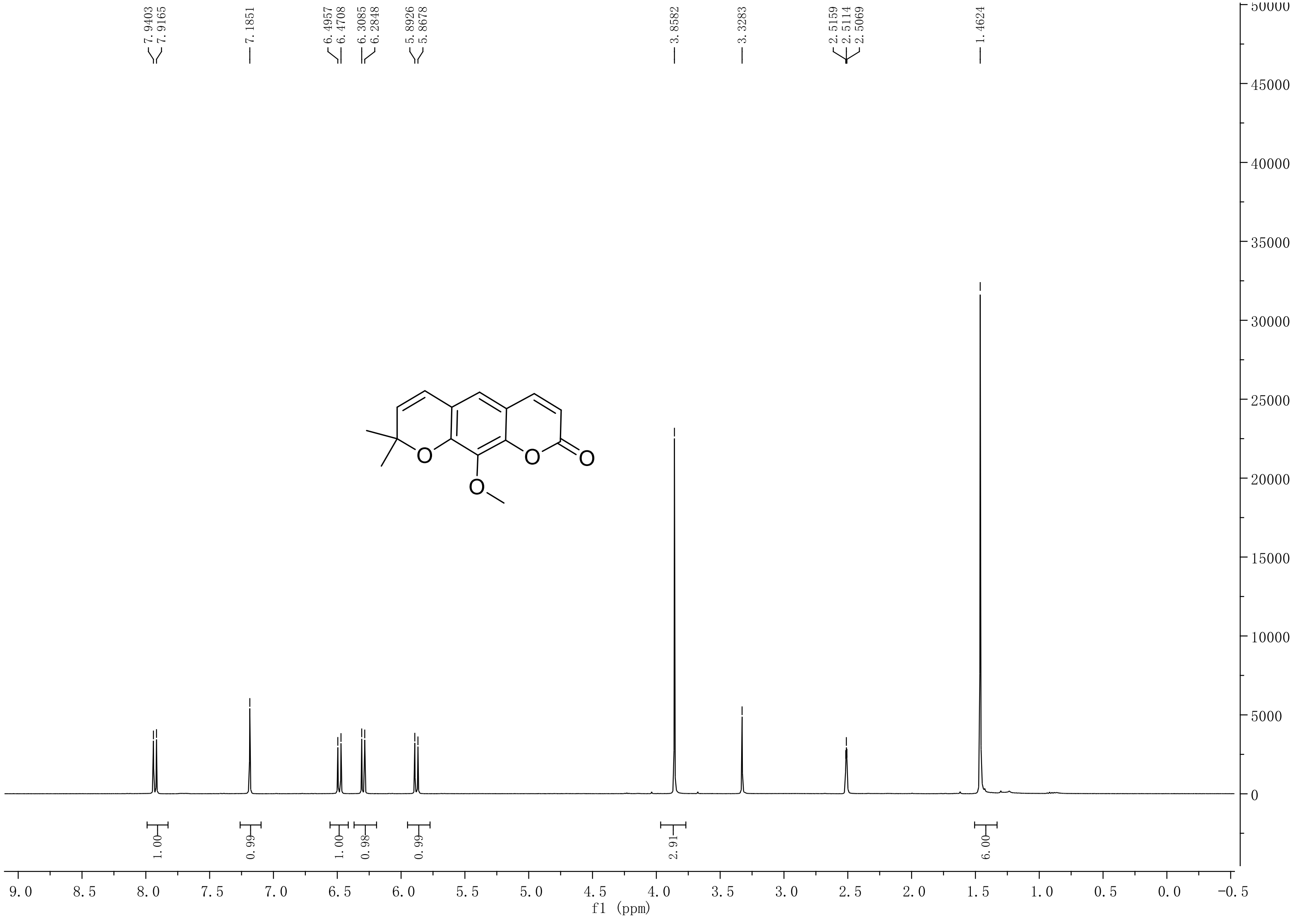

^{13}C NMR spectrum of compound 2 (DMSO-d_6):

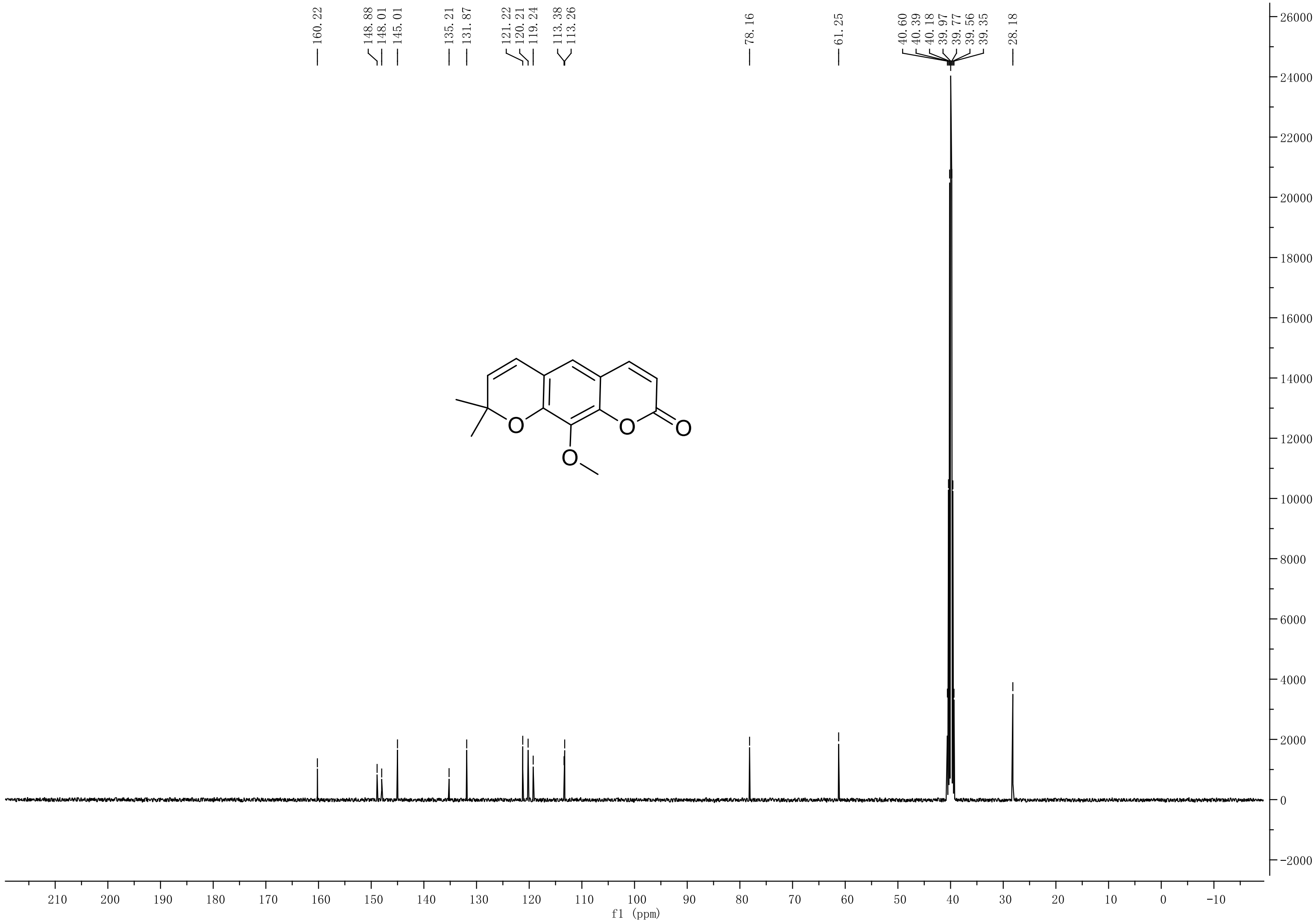

^{1}H NMR spectrum of compound 2 ($CDCl_3$):

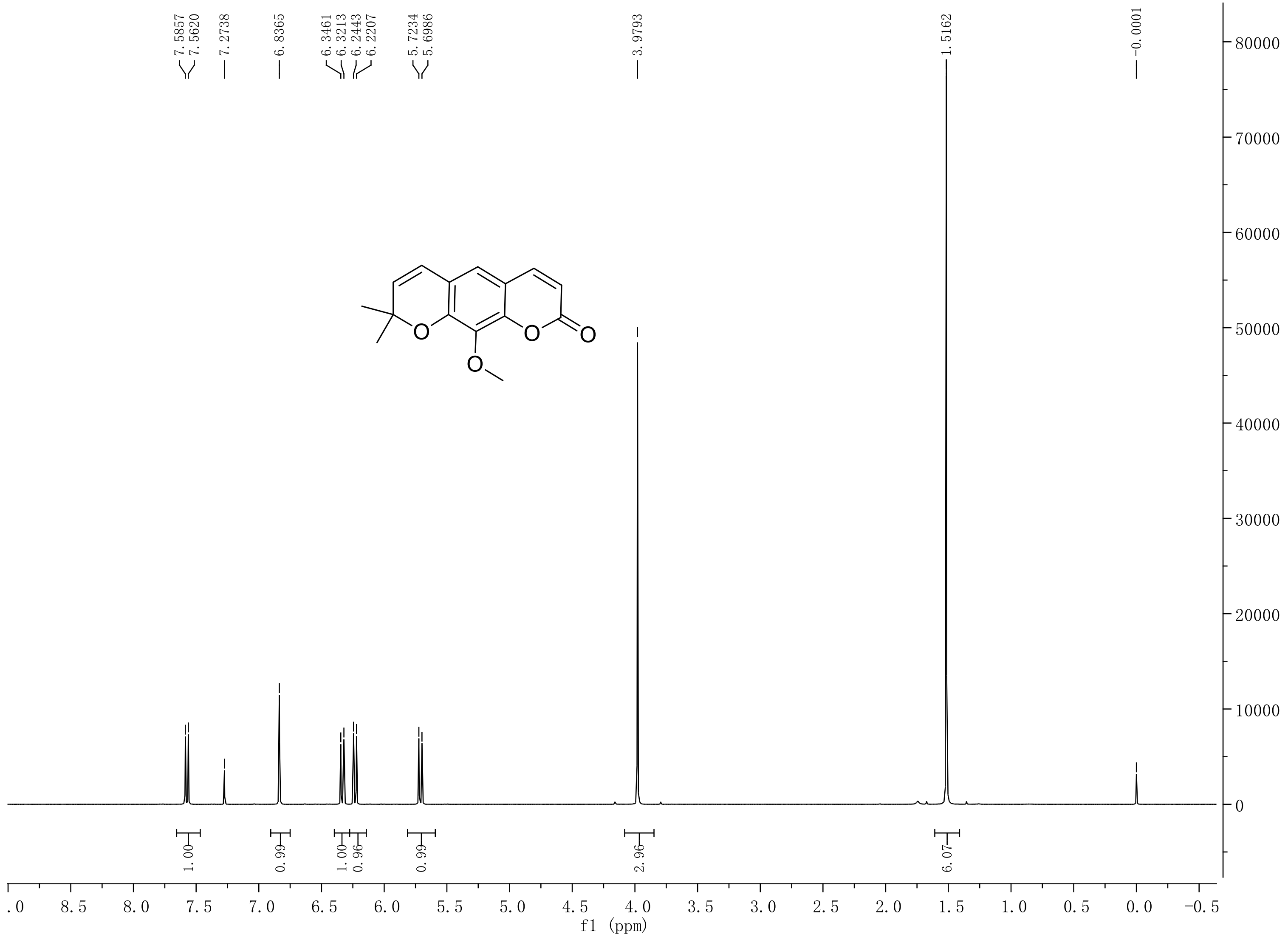

^{13}C NMR spectrum of compound 2 ($CDCl_3$):

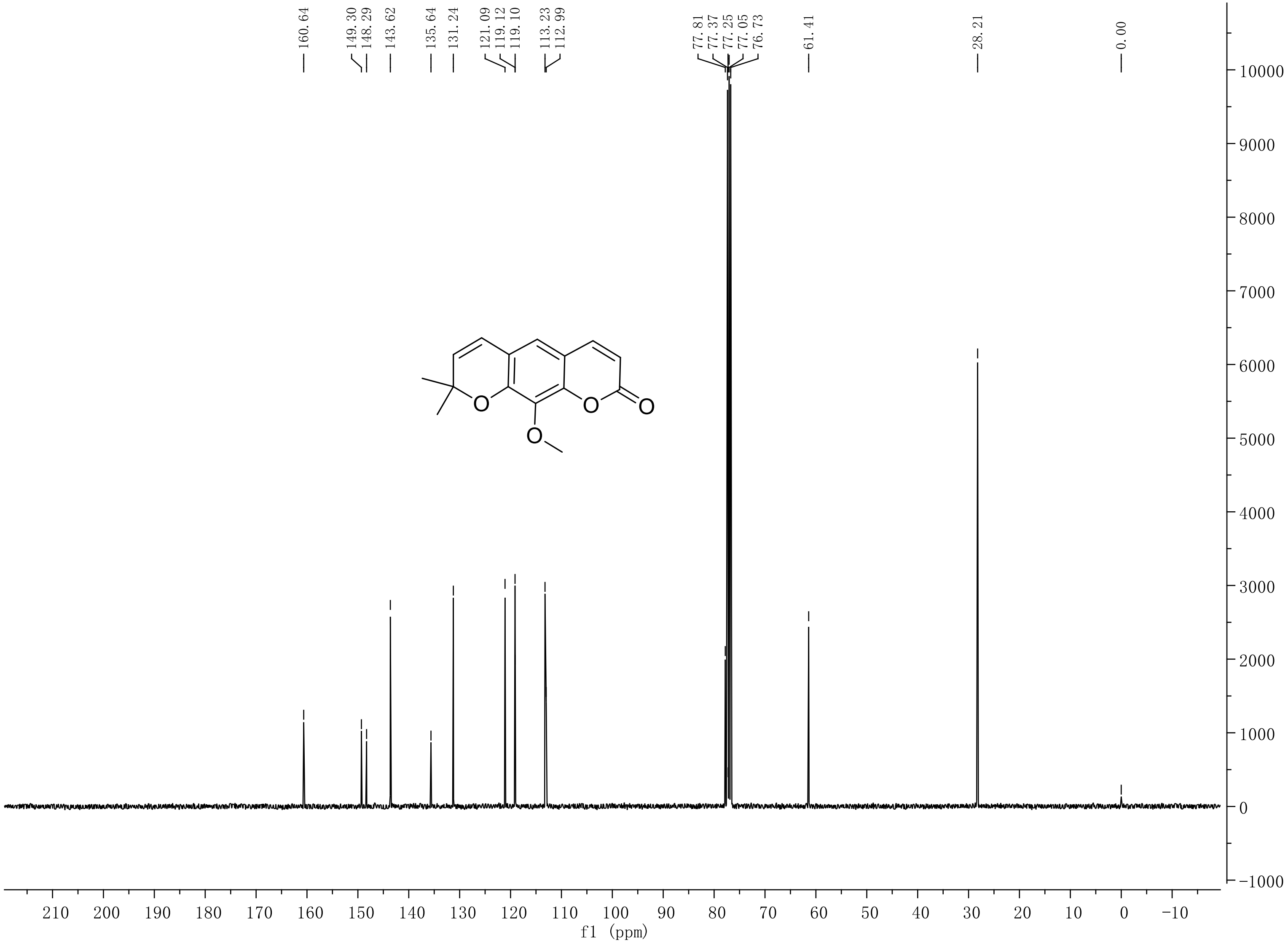

HRMS spectrum of compound 2:

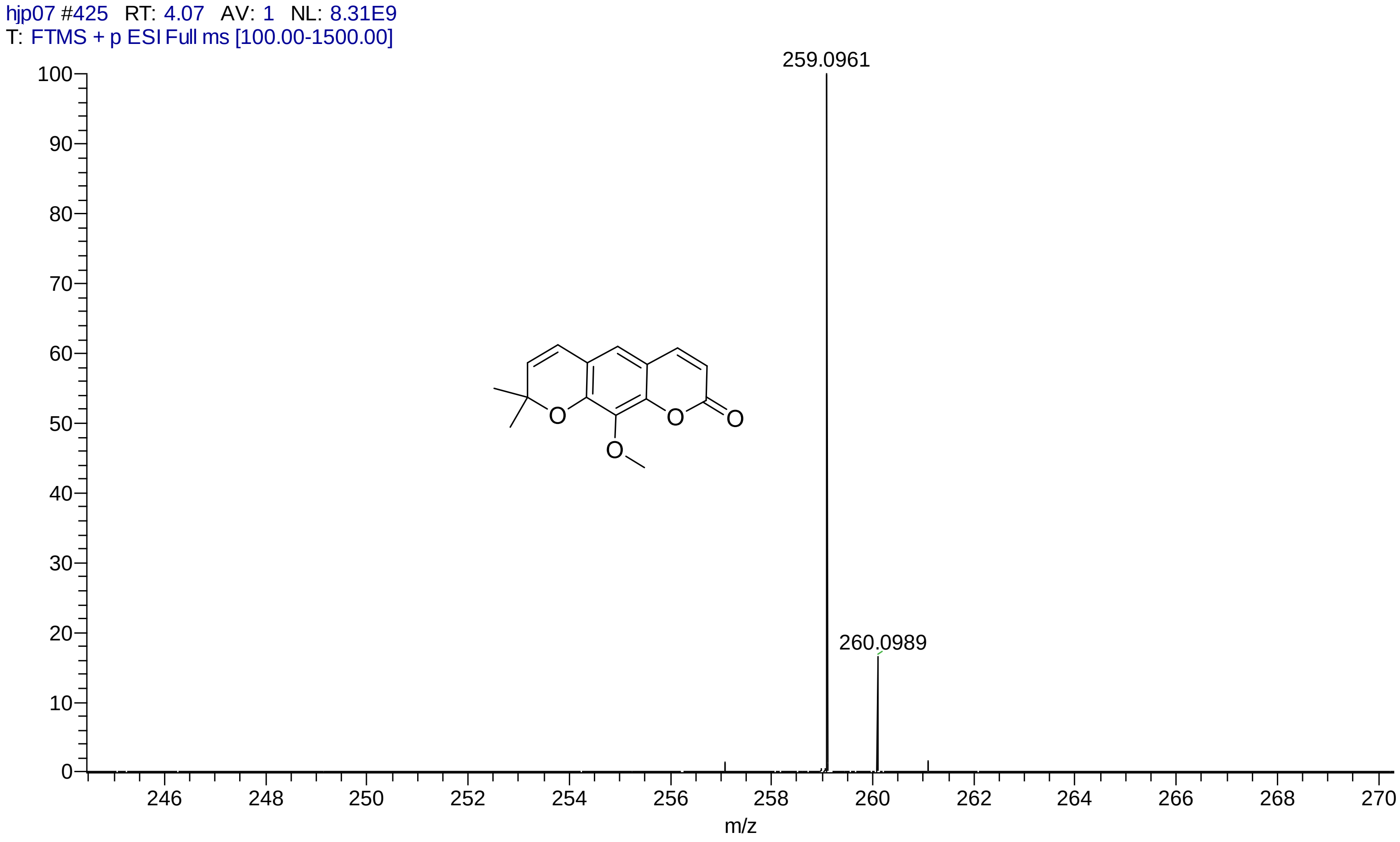

3. Avicennin (compound 3)

^{1}H NMR (400 MHz, DMSO-d_6): δ 8.05 (d, J = 9.6 Hz, 1H), 7.30 (d, J = 16.4 Hz, 1H), 6.61 (dd, J = 16.4, 10.0 Hz, 2H), 6.37 (d, J = 9.6 Hz, 1H), 5.87 (d, J = 10.0 Hz, 1H), 5.15 (s, 2H), 3.75 (s, 3H), 1.97 (s, 3H), 1.47 (s, 6H).

^{1}H NMR (400 MHz, $CDCl_3$): δ 8.04 (d, J = 9.6 Hz, 1H), 7.41 (d, J = 16.4 Hz, 1H), 6.75 (d, J = 16.4 Hz, 1H), 6.63 (d, J = 10.0 Hz, 1H), 6.28 (d, J = 9.6 Hz, 1H), 5.68 (d, J = 10.0 Hz, 1H), 5.19 (d, J = 1.4 Hz, 1H), 5.13 (s, 1H), 3.79 (s, 3H), 2.04 (s, 3H), 1.51 (s, 6H).

^{13}C NMR (101 MHz, DMSO-d_6): δ 160.07, 157.22, 152.51, 149.61, 142.78, 138.81, 136.01, 130.66, 118.49, 117.77, 116.12, 113.50, 110.98, 110.95, 106.25, 78.59, 62.29, 40.48, 27.99, 18.44.

^{13}C NMR (101 MHz, $CDCl_3$): δ 160.89, 157.30, 152.79, 149.43, 143.08, 138.48, 136.52, 129.38, 117.86, 117.25, 116.56, 112.98, 111.74, 110.91, 106.40, 78.01, 77.38, 61.64, 28.14, 18.45.

HRMS: calcd for $C_{20}H_{20}O_4$ $[M+H]^+$: 325.1434, found 325.1431.

^{1}H NMR spectrum of compound 3 (DMSO-d_6):

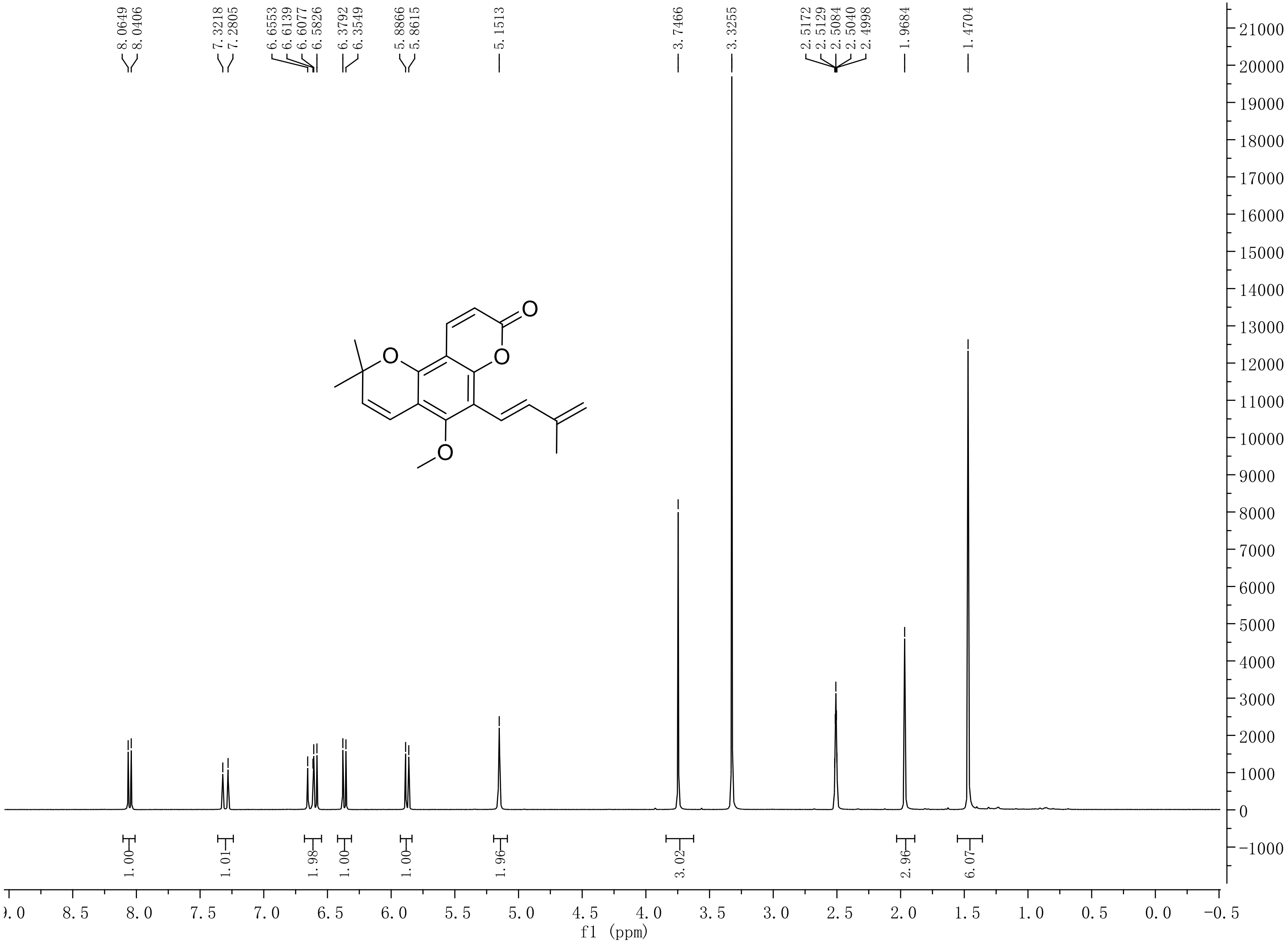

^{13}C NMR spectrum of compound 3 (DMSO-d_6):

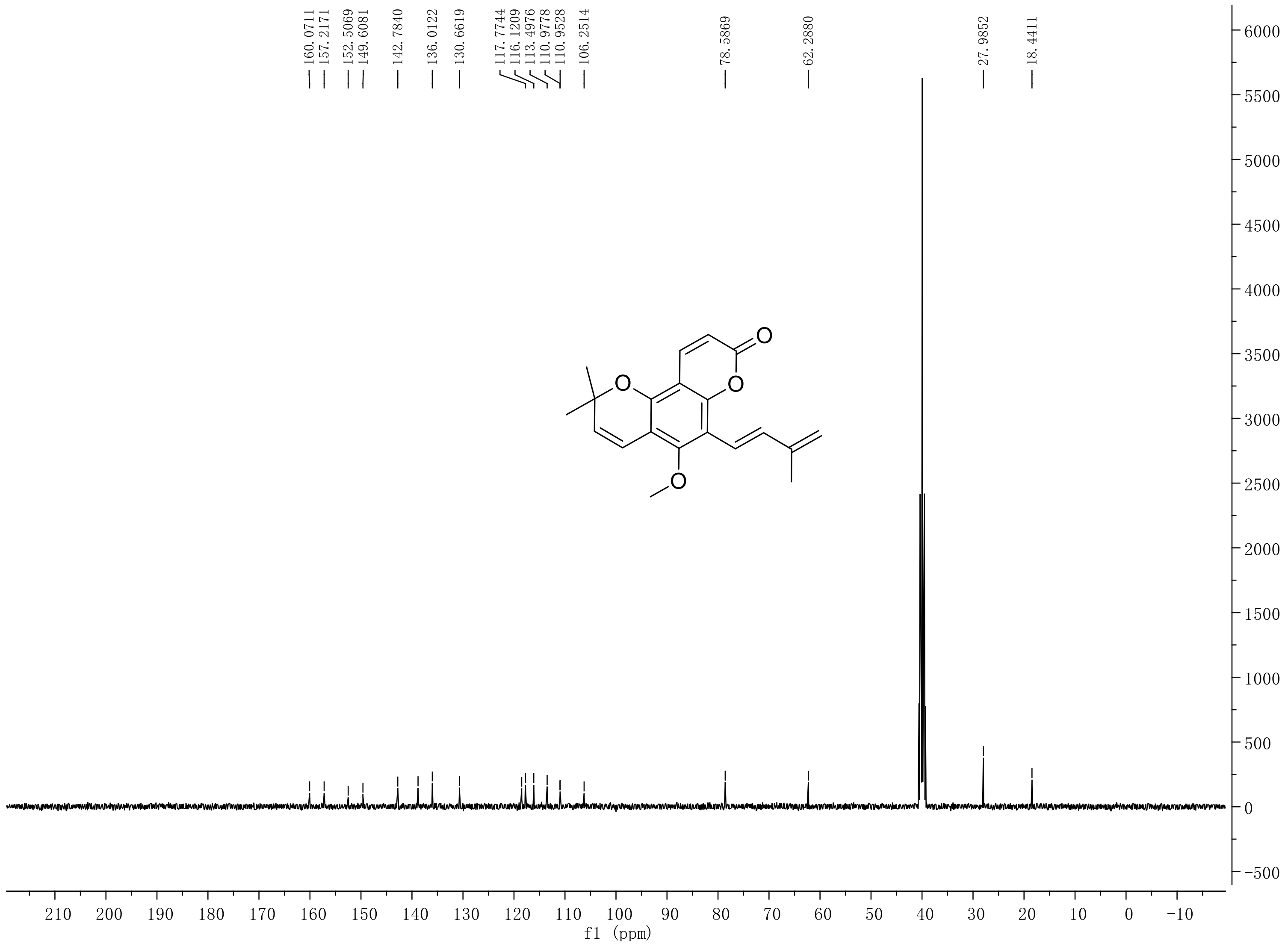

^{1}H NMR spectrum of compound 3 ($CDCl_3$):

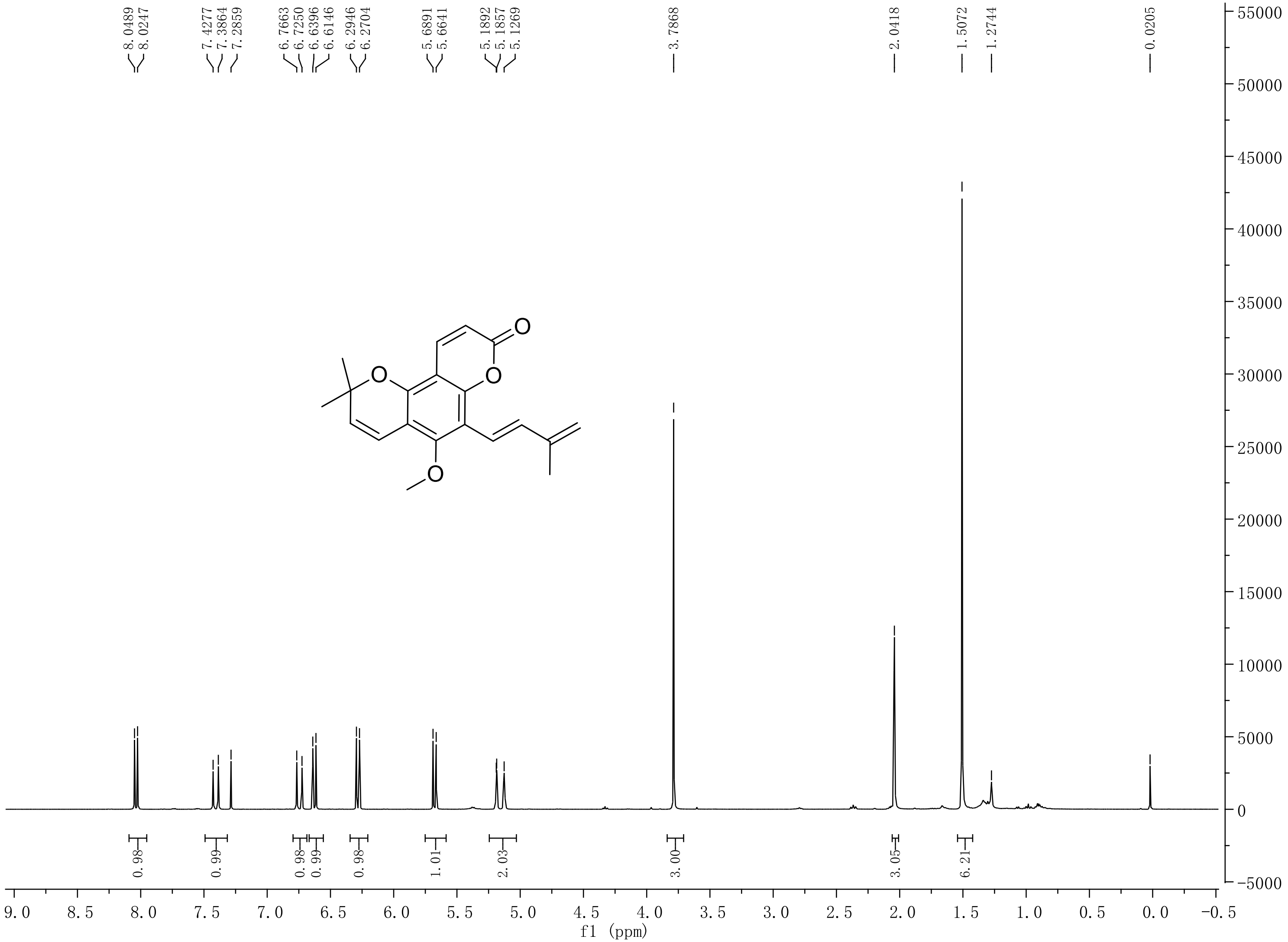

^{13}C NMR spectrum of compound 3 ($CDCl_3$):

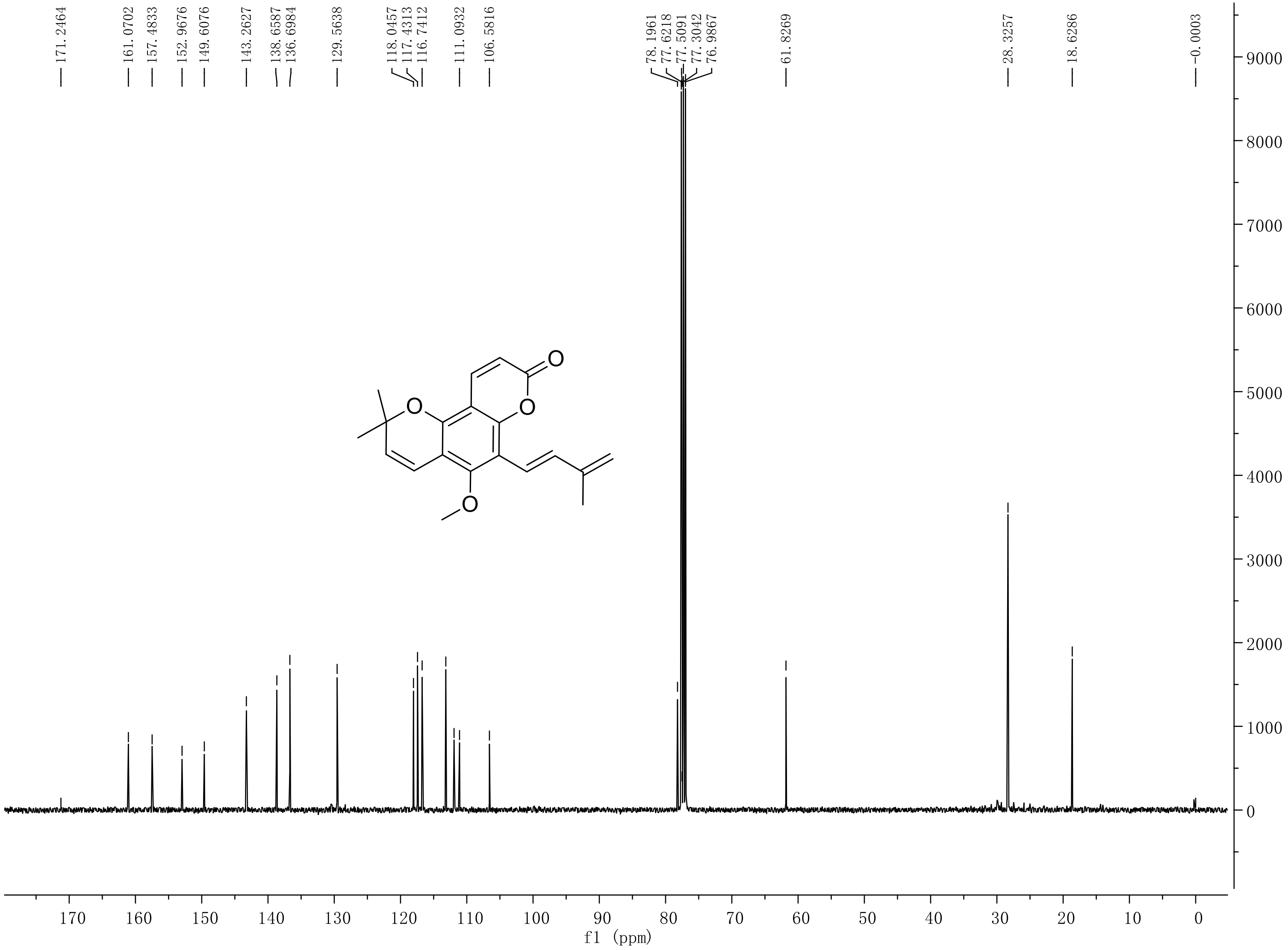

HRMS spectra of compound HJP03:

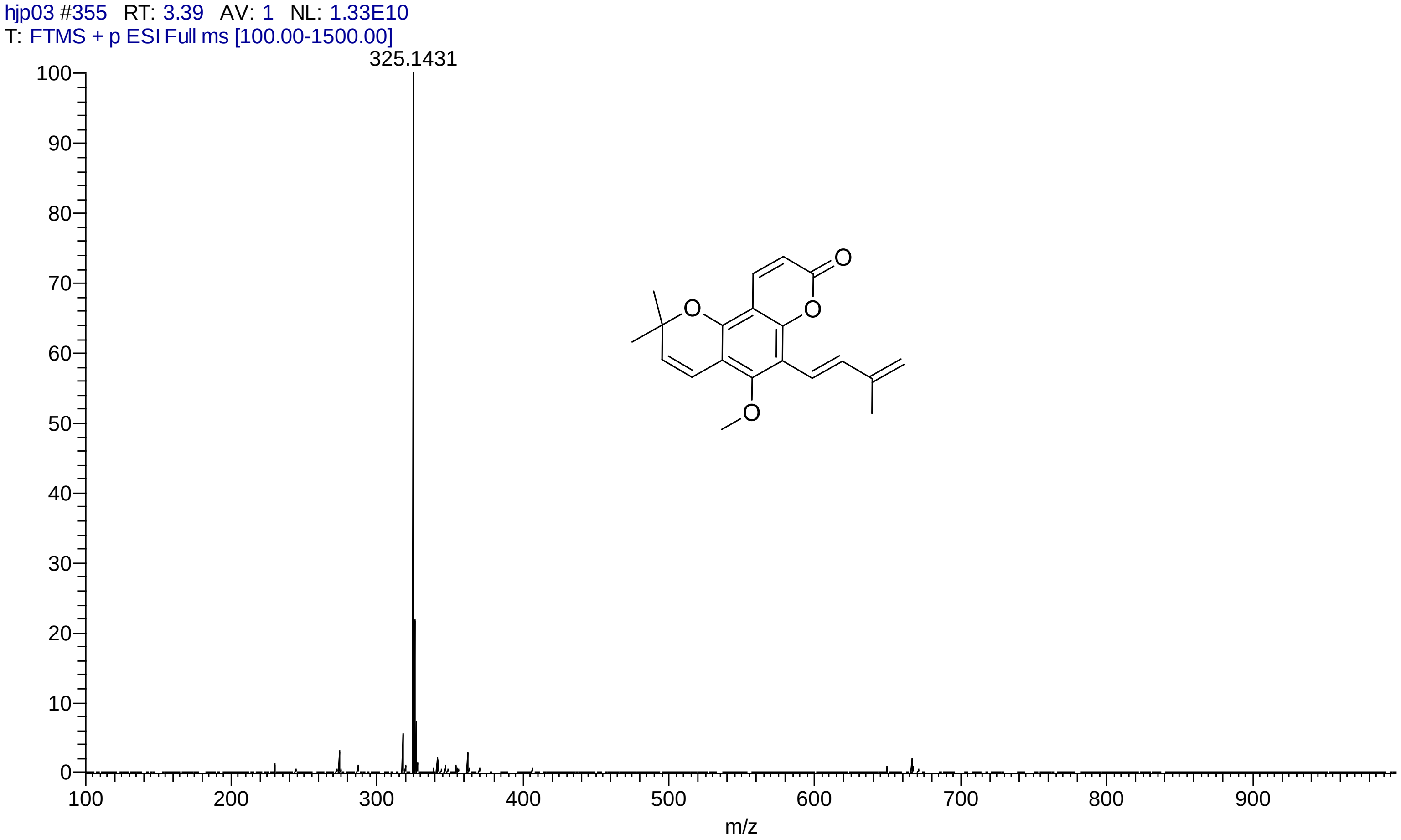

4. Crystallographic data for luvangetin (compound 2): triclinic, space group P-1, a = 7.441 (3) Å, b = 8.365 (4) Å, c = 11.583 (4) Å, α= 75.736 (8)°, β = 80.926 (6)°, γ = 63.596(5)°, V = 624.9 (4) $Å^3$, Z = 2, T = 273 (2) K,μ(Mo)=0.100 mm^{-1}, D calcd. = 1.373 Mg/m^3, 12 124 reflections measured (2.774 ≤ 2Θ ≤ 25.499°), 2290 unique (R(int) = 0.0341) which were used in all calculations. The final R 1 was 0.0632, (I > 2 sigma (I)) and WR 2 was 0.1596. Crystallographic data have been deposited with the Cambridge Crystallographic Data Centre, and the deposition number was KEDFAA 119170.

The X-ray crystal structure and crystal packing of compound 2:

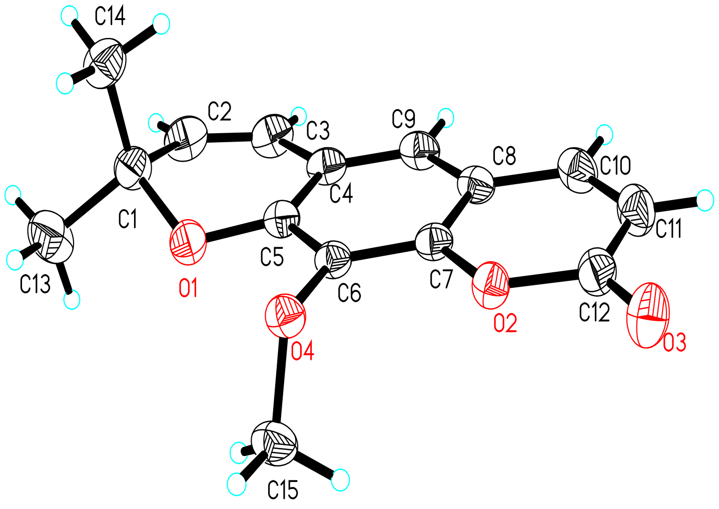

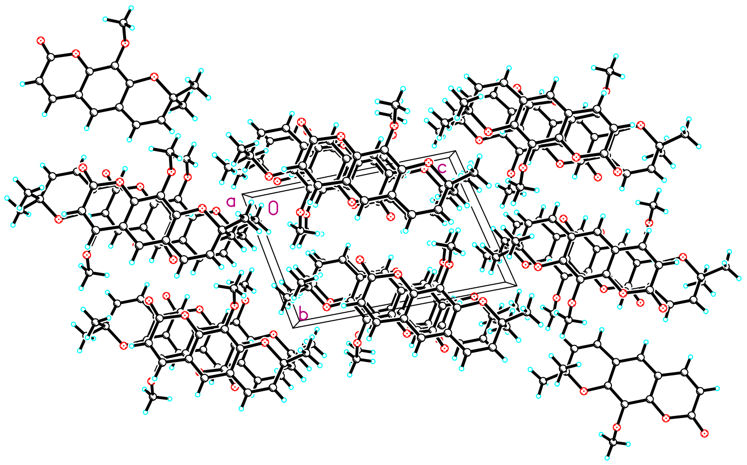

Supplement: Supplementary file 1 [file molecules-24-04207-s001.pdf]
